# Complement 3a Receptor 1 on Macrophages and Kupffer cells is not required for the Pathogenesis of Metabolic Dysfunction-Associated Steatotic Liver Disease

**DOI:** 10.1101/2024.06.26.24309550

**Published:** 2024-06-28

**Authors:** Edwin A. Homan, Ankit Gilani, Alfonso Rubio-Navarro, Maya Johnson, Eric Cortada, Renan Pereira de Lima, Lisa Stoll, James C. Lo

**Affiliations:** 1 Division of Cardiology, Department of Medicine, Cardiovascular Research Institute, Weill Center for Metabolic Health, Weill Cornell Medicine, New York, New York, 10021

**Keywords:** obesity, hepatic steatosis, steatohepatitis, C3aR1, macrophage, Kupffer cell

## Abstract

Together with obesity and type 2 diabetes, metabolic dysfunction-associated steatotic liver disease (MASLD) is a growing global epidemic. Activation of the complement system and infiltration of macrophages has been linked to progression of metabolic liver disease. The role of complement receptors in macrophage activation and recruitment in MASLD remains poorly understood. In human and mouse, *C3AR1* in the iver is expressed primarily in Kupffer cells, but is downregulated in humans with MASLD compared to obese controls. To test the role of complement 3a receptor (C3aR1) on macrophages and liver resident macrophages in MASLD, we generated mice deficient in C3aR1 on all macrophages (C3aR1-MφKO) or specifically in liver Kupffer cells (C3aR1-KpKO) and subjected them to a model of metabolic steatotic liver disease. We show that macrophages account for the vast majority of *C3ar1* expression in the liver. Overall, C3aR1-MφKO and C3aR1-KpKO mice have similar body weight gain without significant alterations in glucose homeostasis, hepatic steatosis and fibrosis, compared to controls on a MASLD-inducing diet. This study demonstrates that C3aR1 deletion in macrophages or Kupffer cells, the predominant liver cell type expressing *C3aR1*, has no significant effect on liver steatosis, inflammation or fibrosis in a dietary MASLD model.

## Introduction

Obesity and related metabolic diseases such as type 2 diabetes (T2D) and metabolic dysfunction-associated steatotic liver disease (MASLD) remain a worldwide epidemic with increasing prevalence^1,2^. MASLD describes the constellation of hepatic lipid deposition, inflammation, and fibrosis associated with obesity and T2D that ultimately leads to MASH cirrhosis, which has become the leading cause of liver transplantation in the United States^3–6^. Notably, MASLD is increasingly recognized as an important risk-enhancing factor for atherosclerotic cardiovascular disease^7,8^.

Liver macrophages help to maintain hepatic homeostasis and consist of embryo-derived resident macrophages called Kupffer cells, which self-renew and do not migrate, or peripheral monocyte-derived macrophages, which infiltrate into liver tissue upon metabolic or toxic liver injury and under certain circumstances can take on Kupffer cell-like identity^9–13^. In obesity, bone marrow-derived myeloid cells migrate to the steatotic liver, and pro-inflammatory recruited macrophages are postulated to drive the progression of MASLD to MASH^14^. Spatial proteogenomics reveals a population of lipid-associated macrophages near bile canaliculi that is induced by local lipid exposure and drives fibrosis in steatotic regions of murine and human liver^15^. In addition, deep transcriptomic profiling in human MASLD has identified candidate gene signatures for steatohepatitis and fibrosis with possible therapeutic implications^16^.

Activation of the body’s complement system leads to increased cell lysis, phagocytosis, and inflammation^17^, and it is increasingly recognized as an important contributor to regulation of metabolic disorders such as T2D and MASLD^18,19^. In human liver biopsies, higher lobular inflammation scores correlate with activation of the complement alternative pathway^20^, which can signal *via* the C3a receptor 1 (C3aR1), a G_i_-coupled G protein-coupled receptor^21^. The complement 3 polypeptide (C3) is cleaved by C3 convertase to the activated fragment, C3a, which then binds C3aR1^22^. Complement factor D (CFD), also known as the adipokine adipsin, is the rate-limiting step in the alternative pathway of complement activation^23,24^.

Several studies have reported opposing roles of adipsin and C3aR1 on hepatic steatosis in diet-induced obesity^25–27^. Our lab has found that adipsin/CFD is critical for maintaining pancreatic beta cell mass and function^28,29^. Murine obese and diabetic models such as *db/db* mice and high fat diet (HFD) feeding result in very low circulating adipsin^23^. Replenishing adipsin in *db/db* mice raises levels of C3a and insulin, lowers blood glucose levels, and inhibits hepatic gluconeogenesis^28^. However, whole-body deletion of C3aR1 decreases macrophage infiltration and activation in adipose tissue, protects from HFD-induced obesity and glucose intolerance, and decreases hepatic steatosis and inflammation^30^. In a model of fibrosing steatohepatitis, bone marrow-derived macrophages were found to activate hepatic stellate cells, which was blunted in whole-body C3aR1 KO mice^31^.

In the present study we aim to explore the macrophage-specific effect of complement receptor signaling in MASLD pathogenesis. To determine the consequences of macrophage and Kupffer cell ablation of C3aR1, we use a murine dietary model of MALFD/MASH, the Gubra Amylin Nash (GAN) diet, which has macronutrient similarities to the Western diet and produces similar histologic and transcriptomic changes to human MASLD/MASH^32–34^.

## Results

### C3AR1 is expressed in human and mouse liver, primarily in Kupffer cells.

**Figure 1.**
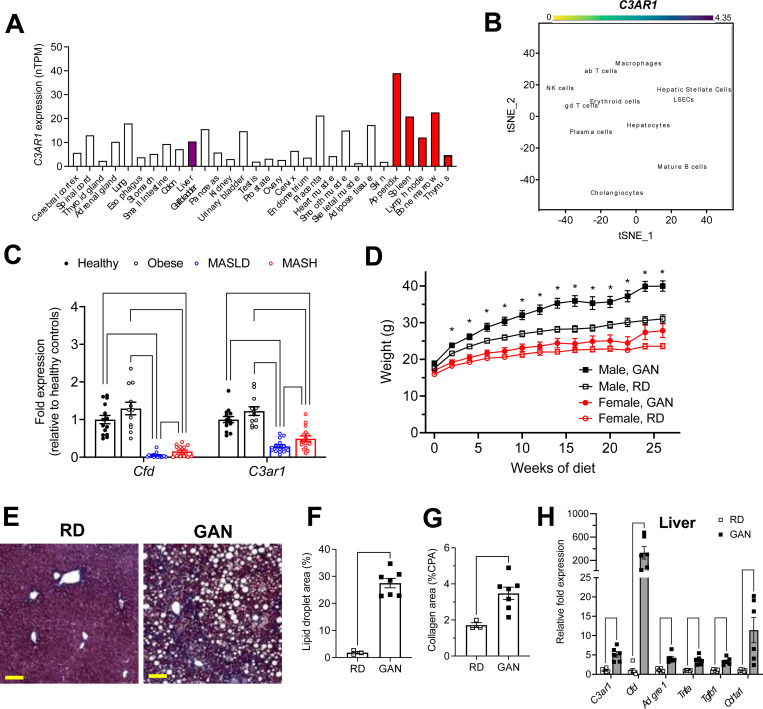
C3AR1 is found in macrophages, is modulated by MASLD/MASH in humans, and is induced by a murine dietary model of MASH. A) Relative *C3AR1* human tissue expression level by tissue, derived from deep sequencing of the mRNA combined dataset (HPA and GTEx) in the Human Protein Atlas, shown as normalized transcripts per million (nTPM). Liver is highlighted in purple and immunologic tissues are highlighted in red. B) Single-cell RNA sequencing distribution of *C3AR1* expression in human liver (tSNE, t-distributed Stochastic Neighbor Embedding). C) Analysis of *CFD* and *C3AR1* expression from liver biopsy samples in patients with MASH, MASLD, obesity without MASLD, and age-matched healthy controls (n = 12–16 per group, Welch *t* test with Holm-Šídák correction for multiple comparisons). D) Weight curve in male and female flox/flox control mice placed on GAN high-fat diet compared to regular diet (RD) controls (males, n = 7; females, n = 6). E) Representative liver section staining by Masson’s Trichrome in male control mice on RD or GAN diet for 28 weeks (scale bar = 100 ∝m). F) Lipid droplet area quantification in liver sections from male control mice, excluding vessel lumens (RD, n = 3; GAN, n = 7). G) Collagen area quantification in liver sections of male control mice (RD, n = 3; GAN, n = 7). H) Gene expression of key macrophage or fibrosis genes in male control mice on GAN or RD (n = 6 per group). Unpaired two-tailed Student’s *t* test (Except 1C as above). Annotations: *, p < 0.05; **, p < 0.01; ***, p < 0.001

In the scRNA-Seq database, Human Protein Atlas, *C3AR1* is broadly expressed throughout the body, with increased abundance in tissues rich in immunologic cell types, such as bone marrow and appendix (Fig. 1A)^35^. In a single-cell transcriptomic database of healthy human liver, *C3AR1* expression predominates in the macrophage and Kupffer cell population, with minimal-to-undetectable *C3AR1* expression in hepatocytes or hepatic stellate cells by scRNA-Seq (Fig. 1B)^36^. In the mouse liver scRNA-Seq database, Tabula Muris, *C3ar1* is similarly expressed primarily in Kupffer cells (Fig. S1)^37^.

### Hepatic CFD and C3AR1 are downregulated in human MASLD/MASH.

We also examined data from Suppli and coworkers, who performed bulk transcriptomic analysis of human liver samples from an age-matched cohort of healthy controls and obese controls without MASLD, as well as MASLD and MASH patients without cirrhosis^38^. Both *CFD and C3AR1* were unchanged in obese subjects without MASLD compared to healthy controls, but both *CFD* and *C3AR1* were significantly downregulated in liver biopsies from both MASLD and MASH patients compared to both healthy controls and obese subjects without MASLD (Fig. 1C). Interestingly, both *CFD* and *C3AR1* levels were slightly higher in MASH individuals compared to those with MASLD only.

### Murine MASH model recapitulates key features of human MASH

At 5 weeks of age, we subjected *C3ar1* flox/flox control mice to standard regular diet (RD) or GAN diet l^32,33^. After 28 weeks of GAN diet, male mice gained body weight compared to RD (Fig. 1D), primarily as fat mass (Fig. S2–3), but weight gain in female GAN-fed mice was attenuated. Histologic signs of MASLD were present in GAN-fed mice (Fig. 1E), most notably hepatic steatosis and hepatocyte ballooning (Fig. 1F), and liver fibrosis measured by collagen deposition nearly doubled with GAN compared to RD (Fig. 1G). Both hepatic *C3ar1* and *Cfd* gene expression were robustly increased on GAN compared to RD, as were markers of macrophage infiltration, hepatic inflammation, and fibrosis, including collagen gene expression, indicating progression to fibrotic MASH (Fig. 1H).

### Macrophage-specific C3aR1 deletion does not alter glucose homeostasis.

**Figure 2.**
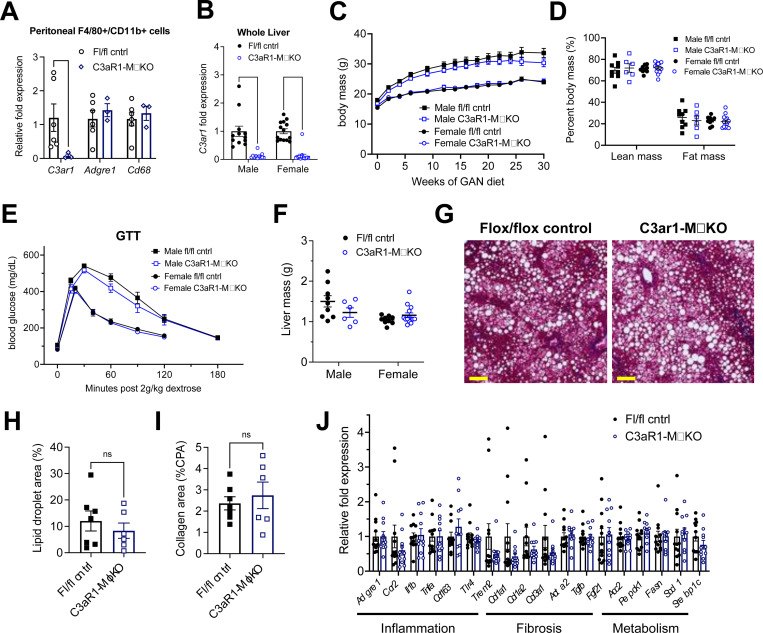
C3aR1 deletion in all macrophages does not affect weight gain, glucose homeostasis, liver steatosis or fibrosis. A) Expression of *C3ar1* in isolated peritoneal F4/80+/CD68+ cells from flox/flox control mice (n = 6) or C3aR1-MφKO male mice (n = 3). B) Expression of *C3ar1* in whole liver from control or C3aR1-MφKO mice (n = 11–12 per male group, n = 13–14 per female group). C) Body mass curve of control or C3aR1-MφKO mice on GAN high-fat diet starting at 5 weeks of age (n = 11–12 per male group, n = 14 per female group). D) Body composition analysis by EchoMRI in control or C3aR1-MφKO mice after 30 weeks GAN diet (n = 6–9 per male group, n = 9–13 per female group). E) Glucose tolerance test in control or C3aR1-MφKO mice with 14h fast after 28 weeks GAN diet (n = 6–9 per male group, n = 9–14 per female group). F) Liver mass in control or C3ar1-MφKO male mice at time of euthanasia after 30 weeks GAN diet (n = 6–9 per male group, n = 9–14 per female group). G) Representative liver section staining by Masson’s Trichrome in male control or C3ar1-MφKO mice (scale bar = 100 ∝m). H) Lipid droplet area in liver sections from male control or C3ar1-MφKO mice, excluding vessel lumens (n = 6–7 per group). I) Collagen area in liver sections from male control or C3ar1-MφKO mice (n = 6–7 per group) J) Relative fold expression of key gene markers for fibrosis, inflammation, and liver metabolism in whole liver from male control or C3ar1-MφKO mice mice after 30 weeks GAN diet (n = 11–12 per group). Unpaired two-tailed Student’s *t* test: Student’s *t* test: *, p < 0.05.

Owing to the differential regulation of the *C3AR1* gene in MASLD between mice and humans, we generated transgenic mice with macrophage-specific deletion of C3aR1 (C3aR1-MφKO) to target both liver resident macrophages and recruited monocytes. Successful deletion of *C3ar1* in macrophages from the C3aR1-MφKO mouse was confirmed by quantitative RT-PCR of isolated peritoneal macrophages that were F4/80+ and CD68+ by fluorescence-activated cell sorting (Fig. 2A). In liver tissue, *C3ar1* expression was reduced by ~88% in both male and female C3aR1-MφKO (Fig. 2B). These results indicate that macrophages account for the vast majority of *C3ar1* expression in the liver.

When placed on GAN diet, there was no significant difference in weight gain between control and C3aR1-MφKO mice (Fig. 2C). There was similarly no difference in percent lean or fat mass between these mice (Fig. 2D). Glucose tolerance tests performed in fasted mice after 27 weeks GAN diet found no significant differences between control and C3aR1-MφKO mice (Fig. 2E). There was also no difference in insulin sensitivity as measured by insulin tolerance tests in male mice (Fig. S4). Insulin resistance as measured by comparing the ratio of fasting glucose level to fasting insulin level (HOMA-IR) was also unchanged between controls and C3aR1-MφKO mice (Fig. S5). Circulating serum ALT levels were unchanged in male control and C3aR1-MφKO mice on GAN diet (Fig. S6).

### Macrophage-specific C3aR1 deletion does not significantly impact hepatic steatosis or fibrosis.

Liver samples collected after 28–30 weeks of GAN or regular diet did not show significant differences in liver mass between control and C3aR1-MφKO mice (Fig. 2F). Male mice on GAN diet developed similar qualitative appearance on histology (Fig. 2G), and slide image analysis showed similar proportions of lipid droplet area and collagen area (Figs. 2H, 2I). This indicates that there were no significant differences in steatosis or fibrosis between GAN-fed control and C3aR1-MφKO male mice. While *C3ar1* expression was markedly reduced in the C3aR1-MφKO liver tissue (Fig. 2B), there were no detectable gene expression changes in markers of fibrosis, inflammation, or lipid handling (Fig. 2J). Similarly, in female mice there were also no significant differences between control and C3aR1-MφKO mouse liver in a subset of key gene markers of fibrosis or inflammation (Fig. S7).

### Kupffer cell-specific C3aR1 deletion does not alter weight gain or glucose homeostasis.

**Figure 3.**
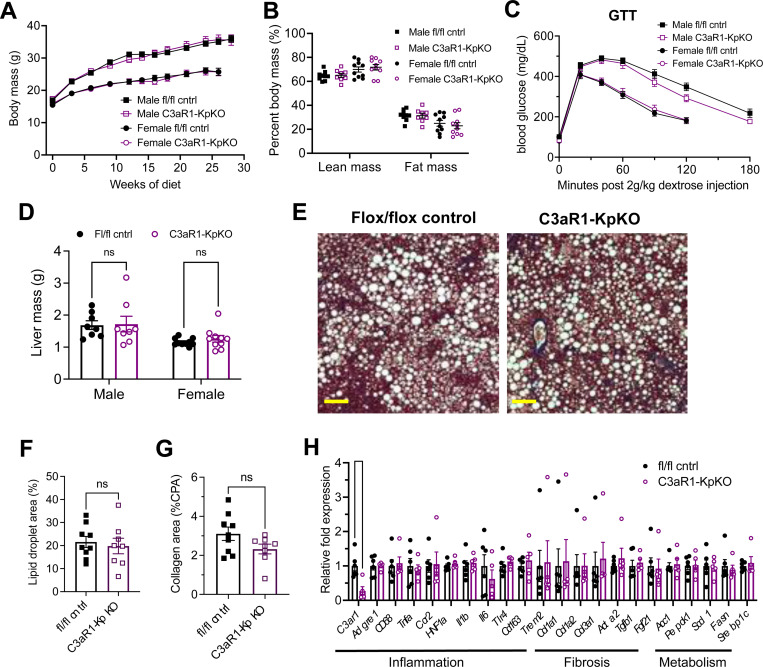
C3aR1 deletion in Kupffer cells does not affect weight gain, glucose homeostasis, liver steatosis or fibrosis. A) Body mass curve on GAN diet in flox/flox control or C3aR1-KpKO mice beginning at 5 weeks of age (n = 8–10 per group). B) Body composition analysis by EchoMRI in control or C3aR1-KpKO mice after 28 weeks GAN diet (n = 8–10). C) Glucose tolerance test in control or C3aR1-KpKO mice with 14h fast after 26 weeks GAN diet (n = 8–10). D) Liver mass in control or C3aR1-KpKO male mice at time of euthanasia after 30 weeks GAN diet (n = 8–10). E) Representative liver section staining by Masson’s Trichrome in control or C3aR1-KpKO male mice (scale bar = 100 ∝m). F) Lipid droplet area quantified on liver sections of control or C3aR1-KpKO male mice, excluding vessel lumens (n = 8–9). G) Collagen area quantified on whole liver section of control or C3aR1-KpKO male mice (n= 8–9). H) Relative gene expression in male control or C3aR1-KpKO mice after 30 weeks GAN diet (n = 5–6). Unpaired two-tailed Student’s *t* test: **, p < 0.01.

To explore whether there may be competing effects between recruited monocytes and liver resident macrophages (Kupffer cells), we next generated Kupffer cell-specific C3aR1 knockout mice (C3aR1-KpKO) and fed them GAN diet. Body weight gain was similar between genotypes for both male and female mice (Fig. 3A), and there was no difference in body composition between control and C3aR1-KpKO mice on GAN diet (Fig. 3B). There was similarly no significant difference in glucose homeostasis between the genotypes during a glucose tolerance test (Fig. 3C).

### Kupffer cell-specific C3aR1 deletion does not significantly impact hepatic steatosis or fibrosis.

Liver mass was not significantly different between control and C3aR1-KpKO mice on GAN diet (Fig. 3D). Liver sections appeared qualitatively similar by histology stained with Masson’s trichrome (Fig. 3E). There were similar levels of hepatic steatosis in these mice as measured by percent lipid droplet area (Fig. 3F). When measured by collagen proportional area, there was no significant differences in liver fibrosis between C3aR1-KpKO and control mice (Fig. 3G). While *C3ar1* expression was reduced by 73% in liver tissue of C3aR1-KpKO mice, there were no significant differences in expression of inflammatory, fibrotic, or lipid handling gene markers (Fig. 3H). *C3ar1* expression similarly decreased by ~90% in liver tissue of female C3aR1-KpKO mice fed regular diet compared to control mice (Fig. S8). These data also indicate that Kupffer cells account for ~80% of hepatic *C3ar1* gene expression in our mouse model of MASLD/MASH.

## Discussion

Overall, we found that macrophage or Kupffer cell expression of *C3ar1* does not impact body weight gain or histologic/transcriptomic features of MASLD/MASH in a murine dietary model. Deletion of C3aR1 in the macrophage population throughout the body, or specifically in Kupffer cells, did not affect weight gain, glucose homeostasis, or extent of hepatic steatosis/fibrosis.

Our findings in macrophage-specific C3aR1 KO mice contrast with prior observations in whole-body C3aR1 KO mice^30^, which are protected from diet-induced obesity, have improved glucose tolerance, and exhibit decreased hepatic steatosis. In both our macrophage- and Kupffer cell-specific C3aR1 KO mice, which had similar degrees of obesity compared to controls, there was no detectable effect on liver steatosis or fibrosis despite the near abrogation of *C3ar1* expression. This raises the possibility that the lower levels of hepatic steatosis and insulin resistance previously observed in the whole body C3aR1 KO mice may be secondary to protection from obesity. Protection from diet-induced obesity in whole-body C3aR1 KO mice may be mediated by a non-macrophage cell type, since our macrophage-specific C3aR1 KO mice were not afforded this protection. The *C3ar1*-expressing cell types that promote obesity and MASLD remains to be determined.

Our laboratory recently reported sex-dependent regulation of thermogenic adipose tissue mediated by adipocyte-derived C3aR1^39^. However, no such sexual dimorphism was observed in hepatic expression of key MASH genes in response to GAN diet in our macrophage- or Kupffer cell-specific C3aR1-deficient mice. Other work has suggested possible compensatory effects from its sister anaphylatoxin receptor C5aR1, with increased cold-induced adipocyte browning and attenuated diet-induced obesity seen in C3aR1/C5aR1 double KO mice^40^.

The strengths of our study include careful metabolic and transcriptomic phenotyping of cell type-specific transgenic mice. Some limitations were our use of a single MASLD dietary model and our focus on the C3aR1 pathway. While the GAN diet recapitulates many features of human MASH due to its similarity to Western diet^34^, relatively low levels of fibrosis were seen in our study, potentially related to initiating the diet at young age; more rapid fibrosis induction has been seen when GAN diet is initiated at older ages^41^. Lastly, while *C3AR1*/*C3ar1* expression is very low in non-macrophage cells (Fig. B, S1), C3aR1 signaling on other hepatic cell types not explored in this study, such as hepatic stellate cells, could mediate the observed effect in the whole-body C3aR1 KO mouse.

Deletion of C3aR1 in macrophages generally, or in liver resident macrophages specifically, had no major effect on systemic glucose homeostasis and hepatic steatosis, inflammation, and fibrosis in this murine dietary model of MASLD/MASH. The complement system is a complex entity directing an important part of the body’s inflammatory and tissue repair response in MASLD. Further work is needed to elucidate the mechanisms of the role of C3aR1 in the pathogenesis of MASH and cirrhosis.

## Materials and Methods

### Animals

*C3ar1 flox/flox* mice were on the C57BL/6J background as described^42^. Homozygous LysM-Cre mice on the C57BL/6J background were purchased from Jackson Laboratories (Strain #004781). *C3ar1 flox/flox* homozygous mice were used in the experiments as controls from the same backcross generation^39^. All mice were maintained in plastic cages under a 12h/12h light/dark cycle at constant temperature (22°C) with free access to water and food. Mice were fed regular diet containing 4.5%kcal fat PicoLab Rodent diet 20 (LabDiet) or GAN diet containing 40%kcal HFD (mostly palm oil) with 20% fructose and 2% cholesterol (D09100310, Research Diets) for 28–30 weeks. Fat mass and lean mass were determined via noninvasive 3-in-1 body composition analyzer (EchoMRI). Mice were humanely euthanized with CO_2_ inhalation followed by exsanguination by cardiac puncture.

### Blood chemistry and serum insulin analysis

Mice were fasted overnight (14–16 hours) for glucose tolerance tests and injected intraperitoneally with syringe-filtered D-glucose solution (2g/kg). For insulin tolerance test, mice were fasted for 6 hours and injected with 0.5 mIU/kg insulin. Blood glucose levels were assayed by commercial glucometer (OneTouch) by tail vein blood samples. Plasma insulin levels were measured from mice fasted for 6 hours. Tail vein blood was collected into lithium heparin-coated tubes, centrifuged at 2000xg at 4°C, and plasma insulin levels were determined by ELISA using a standard curve (Mercodia). Serum alanine aminotransferase levels were measured in serum from blood collected via cardiac puncture using a commercially available colorimetric assay (TR71121, ThermoFisher Scientific).

### Peritoneal macrophage isolation and flow cytometry

Peritoneal macrophages were isolated from as previously described^43^. Briefly, mice were euthanized then immediately injected intraperitoneally with 10 mL phosphate-buffered saline (PBS, pH 7.4) at room temperature. After a 3–5 minute incubation period, peritoneal fluid was removed with sterile needle and syringe and placed on ice. After centrifugation at 300xg, the pellet was resuspended in PBS containing 2% fetal bovine serum and 0.1% sodium azide. Cells were stained with phycoerythrin-conjugated anti-F4/80 (clone BM8, cat. #123110) and fluorescein isothiocyanate-conjugated anti-CD11b (clone M1/70, cat. #101206) fluorescent antibodies (Biolegend). Stained cells were loaded on MA900 fluorescence-activated cell sorter (Sony), and dual-positive F480+/CD11b+ cells were sorted for subsequent RNA extraction.

### Histological studies

A mid-distal portion of the left liver lobe was fixed with 10% buffered formalin and transferred to 70% ethanol. Samples were embedded in paraffin, sectioned at ~5μm thickness, and stained with Masson’s trichrome. Slides were imaged using Zeiss Axioscan7 at 20x magnification. Histologic analyses were performed using ImageJ software (version 1.53t). Lipid droplet area was quantified by subtracting non-droplet area in the green channel from total section area of 2–3 independent sections. Collagen proportionate area was quantified by measuring total area in the red channel after reducing intensity threshold to 60–70.

### RNA extraction and real-time quantitative PCR analysis

Total RNA from liver tissue lysates was extracted using Trizol reagent (Invitrogen) followed by RNAeasy Mini kit (Qiagen) as per manufacturer’s protocol. RNA was reverse-transcribed using the High Capacity cDNA RT kit (Thermo). Quantitative PCR was performed using SYBR Green Master Mix (Quanta) and specific gene primers on QuantStudio6 Flex Real-Time PCR Systems (Thermo Fisher Scientific) using the delta-delta Ct method. Expression levels were normalized to Ribosomal protein S18 (*Rps18*). Primer sequences are listed in Supplementary Table A.

### Statistical analyses

All statistical analyses were performed using GraphPad Prism10. Unpaired two-tailed Student’s *t* test with Welch correction for most analyses, with Holm-Šídák correction for multiple comparisons where applicable, and p<0.05 was considered statistically significant.

## Supplementary Material

Supplement 1

## Data Availability

Data will be made available upon reasonable request.
