# Supplementary material for: Complement 3a Receptor 1 on Macrophages and Kupffer cells is not required for the Pathogenesis of Metabolic Dysfunction-Associated Steatotic Liver Disease
Source: medRxiv. 2024 Jun 28:2024.06.26.24309550. Preprint. [Version 1] doi: 10.1101/2024.06.26.24309550 (PMC11230319; doi:10.1101/2024.06.26.24309550)
Supplement: Supplement 1 [file NIHPP2024.06.26.24309550v1-supplement-1.pdf]

medRxiv preprint doi: https://doi.org/10.1101/2024.06.26.24309550; this version posted June 28, 2024. The copyright holder for this preprint (which was not certified by peer review) is the author/funder, who has granted medRxiv a license to display the preprint in perpetuity.
It is made available under a CC-BY 4.0 International license .

Supplementary figures.

S1) Single cell RNA sequencing analysis of *C3ar1* expression in mouse liver tissue (see text).

S2) Percent lean and fat mass of flox/flox control mice after 20 weeks of GAN or RD diet (n = 6-7 per group).

S3) Absolute lean and fat mass of flox/flox control mice after 20 weeks of GAN or RD diet (n = 6-7 per group).

S4) Insulin tolerance test in control or C3aR1-MφKO male mice with 14h fast after 29 weeks GAN diet (n = 6-9 per group).

S5) HOMA-IR measurement of insulin resistance in control or C3aR1-MφKO mice with 6h fast after 27 weeks GAN diet (n = 6-9 per male group, n = 9-13 per female group).

S6) Serum alanine aminotransferase levels in control or C3aR1-MφKO male mice after 30 weeks GAN diet (n = 4 per group).

S7) Relative gene expression in control or C3aR1-MφKO female mice after 30 weeks GAN diet (n = 13-14 per group).

S8) Relative *C3ar1* expression in control or C3aR1-KpKO female mice after 30 weeks RD diet (n = 2-3 per group).

Unpaired two-tailed Student's *t* test: **, $p < 0.01$; ***, $p < 0.001$.

medRxiv preprint doi: https://doi.org/10.1101/2024.06.26.24309550; this version posted June 28, 2024. The copyright holder for this preprint (which was not certified by peer review) is the author/funder, who has granted medRxiv a license to display the preprint in perpetuity.
It is made available under a CC-BY 4.0 International license .

Supplementary Figures.

S1

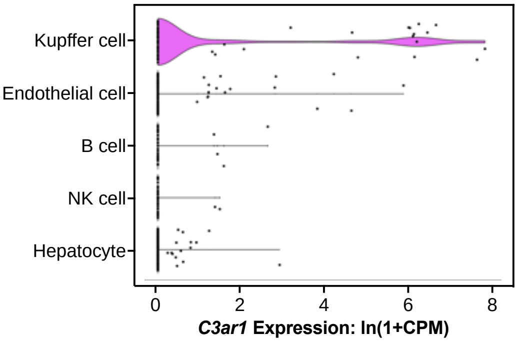

S2

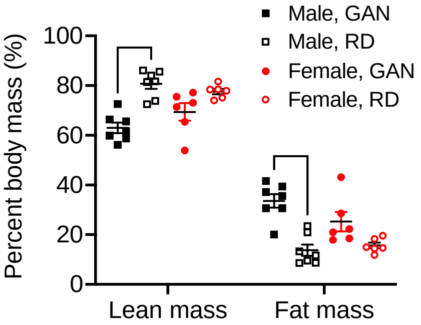

S3

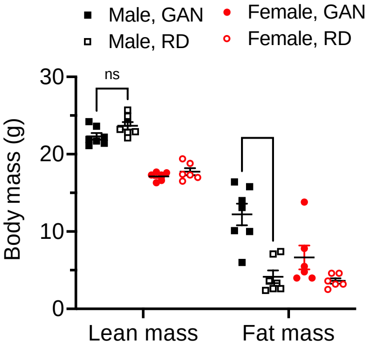

S4

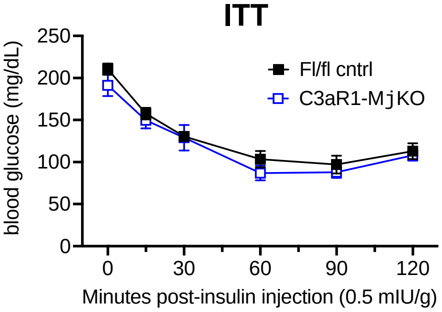

S5

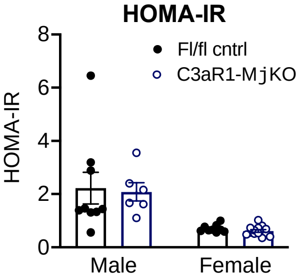

S6

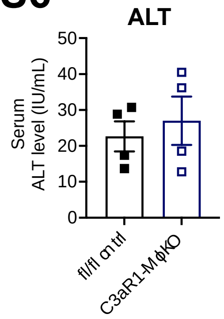

S7

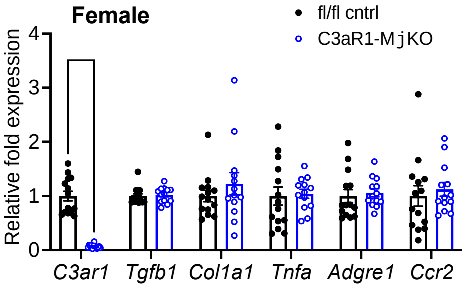

S8

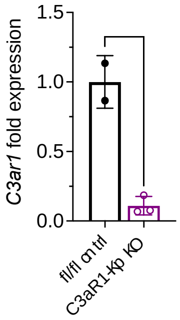

Supplementary figures.

S1) Single cell RNA sequencing analysis of *C3ar1* expression in mouse liver tissue (see text).
S2) Percent lean and fat mass of flox/flox control mice after 20 weeks of GAN or RD diet (n = 6-7 per group).
S3) Absolute lean and fat mass of flox/flox control mice after 20 weeks of GAN or RD diet (n = 6-7 per group).
S4) Insulin tolerance test in control or C3aR1-MjKO male mice with 14h fast after 29 weeks GAN diet (n = 6-9 per group).
S5) HOMA-IR measurement of insulin resistance in control or C3aR1-MjKO mice with 6h fast after 27 weeks GAN diet (n = 6-9 per male group, n = 9-13 per female group).
S6) Serum alanine aminotransferase levels in control or C3aR1-MjKO male mice after 30 weeks GAN diet (n = 4 per group).
S7) Relative gene expression in control or C3aR1-MjKO female mice after 30 weeks GAN diet (n = 13-14 per group).
S8) Relative *C3ar1* expression in control or C3aR1-KpKO female mice after 30 weeks RD diet (n = 2-3 per group).
Unpaired two-tailed Student's *t* test: **, $p < 0.01$; ***, $p < 0.001$.

medRxiv preprint doi: https://doi.org/10.1101/2024.06.26.24309550; this version posted June 28, 2024. The copyright holder for this preprint (which was not certified by peer review) is the author/funder, who has granted medRxiv a license to display the preprint in perpetuity.
It is made available under a CC-BY 4.0 International license .

Supplementary Table S1.

***Mus musculus* gene name**	**Forward qPCR primer**	**Reverse qPCR primer**
Acc2	GCCTCCACTCACATTGGTTT	ATTGAAGAAAGCTGGGCTGA
Acta2	GGCTCTGGGCTCTGTAAGG	CTCTTGCTCTGGGCTTCATC
Adgre1	TGCATCTAGCAATGGACAGC	GCCTTCTGGATCCATTTGAA
C3ar1	TGACAGGTCAGCTCCTTCCT	CATTAGGAGGCTTTCCACCA
Ccr2	ATCCACGGCATACTATCAACATC	CAAGGCTCACCATCATCGTAG
Cd163	TCCACACGTCCAGAACAGTC	CCTTGGAAACAGAGACAGGC
Cfd	CGTACCATGACGGGGTAGTC	ATCCGGTAGGATGACACTCG
Col1a1	GTGCTCCTGGTATTGCTGGT	GGCTCCTCGTTTTCCTTCTT
Col1a2	GCCACCATTGATAGTCTCTCC	CACCCCAGCGAAGAACTCATA
Col3a1	GGGTTTCCCTGGTCCTAAAG	CCTGGTTTCCCATTTTCTCC
Fasn	TTGCTGGCACTACAGAATGC	AACAGCCTCAGAGCGACAAT
Fgf21	CTGCTGGGGGTCTACCAAG	CTGCGCCTACCACTGTTCC
Hnf1a	GACCTGACCGAGTTGCCTAAT	CCGGCTCTTTCAGAATGGGT
Il1b	CTGGTGTGTGACGTTCCCATTA	CCGACAGCACGAGGCTTT
Il6	ACAACCACGGCCTTCCCTACTT	CACGATTTCCCAGAGAACATGTG
Pepck1	TCATCATCACCCAAGAGCAG	CACATAGGGCGAGTCTGTCA
Rps18	CATGCAGAACCCACGACAGTA	CCTCACGCAGCTTGTTGTCTA
Scd1	CGCCCAAGCTGGAGTACGTC	CGCCCAAGCTGGAGTACGTC
Srebp1c	CTGGCAGTTCCATTGACAAG	ACTGAAGCTGGTGACTGCTG
Tgfb1	TGCGCTTGCAGAGATTAAAA	AGCCCTGTATTCCGTCTCCT
Tlr4	TGTCATCAGGGACTTTGCTG	GGACTCTGATCATGGCACTG
Tnfa	ACGGCATGGATCTCAAAGAC	AGATAGCAAATCGGCTGACG
Trem2	CTACCAGTGTCAGAGTCTCCGA	CCTCGAAACTCGATGACTCCTC
